# AGE-VARYING COGNITIVE IMPAIRMENT AND SOCIAL SUPPORT AMONG PEOPLE AGING WITH AND WITHOUT HIV

**DOI:** 10.1093/geroni/igad104.1349

**Published:** 2023-12-21

**Authors:** Alexis Bender, Regine Haardörfer, Molly Perkins, David Moore, Hannah Cooper

**Affiliations:** Emory University, Atlanta, Georgia, United States; Emory University, Atlanta, Georgia, United States; Emory University, Atlanta, Georgia, United States; University of California at San Diego, San Diego, California, United States; Emory University, Atlanta, Georgia, United States

## Abstract

There is compelling but limited evidence that the combination of HIV, methamphetamine (meth) use, and aging form a deleterious trio that undermines cognitive functioning. Currently, a gap exists in our understanding of the intertwining relationships among drug use, HIV, and cognitive function across biological ages and over time. Interventions to reduce meth use and support brain health among aging PWH are vital and improving social relationships is a promising area of intervention. Cross-sectional and limited longitudinal studies indicate that positive social relationships and social support are associated with better cognitive function among healthy older populations. This association has received limited attention among PWH who use drugs beyond adolescence and young adulthood. This study leverages NIH’s prior and ongoing investments in research to develop a new analytic cohort by combining participants from 23 studies at the HIV Neurobehavioral Research program. Enrollment in this cohort spans from 2009-2021. At baseline, our sample consists of 862 people with a mean age of 52 (range 18-81). Most participants are male (73%), White (74%), and over half (61%) are living with HIV. Using a life course perspective and time-varying estimation modeling, we examine age-varying cognitive impairment and variation in cognitive function across age by HIV status, meth use, and social relationships. First, we examine how cognitive impairment changes over time for the full sample then examine the differing transitions/turning points among those aging with and without HIV and meth use. Lastly, we show how positive and negative social relationships alter these trajectories among each group.

